# Learning Effectiveness of Social Work Methods With Groups, in Online and Face-to-Face Contexts

**DOI:** 10.3389/fpsyg.2021.649691

**Published:** 2021-08-17

**Authors:** Nicoleta Neamţu, Cristina Faludi

**Affiliations:** Department of Social Work, Babeş-Bolyai University, Cluj-Napoca, Romania

**Keywords:** effectiveness of online learning, social group work, ecological influence on groups, online vs. face-to-face groups, Romanian students, social work competencies

## Abstract

During the last three decades, thousands of highly qualified social workers who graduated from Romanian universities were employed in the public systems of social work of the European Union. Social group work is studied as a compulsory discipline for undergraduate students. The major focus of our study was the effectiveness of the learning of Social Work Methods with Groups (SWMG) of students, using workshops in a full-time undergraduate program from Romania. We were interested in finding out the perceptions of students about their learning processes and outcomes in the context of teaching the same discipline exclusively in the online medium, due to the pandemic, and in the face-to-face environment *via* traditional classroom instruction. This study had a mainly quantitative design, covering two academic years between 2018 and 2020 for the two cohorts of social work students. The core analysis was focused on the activities of students at the SWMG laboratories: 50 students in 2020 and 92 students in 2019. Descriptive, inferential statistics and thematic content analysis were applied to two types of deliverables of students: the self-assessment sheet and the group plan. The results of our study showed that training of cognitive and self-awareness skills prevailed among the students who learned online in 2020, while the acquisition of interpersonal skills was reported at a significantly higher level by students who learned in the face-to-face medium in 2019. The students in the traditional classrooms favoured the training of other professional skills, too, like problem-solving skills. However, students who studied exclusively online attributed a significantly greater overall usefulness of SWMG workshops for professional practise than their peers who participated in the face-to-face laboratories. A remarkable result was that more therapeutic and support groups were preferred in the online environment, maybe related to the concerns generated by the pandemic. Remote education forced most students to return to their original places of residence, mostly in the countryside and brought negative psychological effects caused by social isolation due to the pandemic. Remote learning is not the most desirable educational option. Students gain most from blended teaching-learning vehicles: face-to-face and online medium.

## Main Themes of the Article

(1) Higher Education (HE) Delivery: Challenges of emergency remote teaching: (1) technology and remote delivery; and (2) assessment limitations arising from online formats.
(2) Reflections on the Past and into the Future: What will be the “new normal” in HE? What values, vulnerabilities, priorities, and opportunities have been revealed in the crisis and our varied responses, realigning relations between teaching, research, and service internationalisation.

## Introduction

Our study was motivated by the new challenges and opportunities from higher social work education created in the context of the COVID-19 pandemic. The prolongation of the COVID 19 crisis forced our university to reconsider the way we approach the teaching-assessment process. To not compromise the quality of the educational act, we needed an *ad hoc* reconceptualisation of the teaching–learning–evaluation activities. More precisely, in this new context, developing and implementing novel teaching strategies was necessary. They capitalise on the strengths and good practises extracted from classical teaching, which were adapted to the constraints of online education.

The relocated teaching during the state of emergency, determined by the pandemic, was overcome through meeting the quality standards of updated digitised teaching. More precisely, an optimised teaching strategy, namely a so-called interactive online teaching, was proposed. This approach highlights the key role of student interactions (interactions with informational content with teachers and colleagues, respectively) and will formulate concrete suggestions on optimising these interactions.

These types of interaction are the essence of student-centred teaching. The focus was mainly on those technological features that superiorly favour the use of strategies and teaching methods that increase the quality of teaching-learning. The elements that individualise this didactic paradigm are synthetically exposed in the following, highlighting innovative elements: (a) from a psycho-pedagogical perspective, it is based on social-constructivist conceptualisation; (b) it assimilates actively and selectively the requirements of online education; (c) it promotes flexibility regarding the sharing and alternation of synchronous and asynchronous activities; and (d) it explicitly focuses on teaching strategies that increase the level of student interaction with informational content and, respectively, the interactions of students with teachers and colleagues.

The designed solutions optimised both didactic versions that Babeş-Bolyai University has undertaken for the first and, respectively, for the second semester of the academic year, during the pandemic: predominantly or exclusively online and, respectively, hybrid or blended learning.

This redesign of instructional tactics at the university level focused on all key components of educational acts and, respectively, of the context in which they occur: teaching, assessment, feedback, learning community, educational climate, and issues related to persistence and the motivation students for learning.

In the specific context presented above, we were particularly concerned about the effectiveness of learning Social Work Methods with Groups (SWMG) among students, using workshops in an undergraduate program from the Romanian university where we are employed as teaching professionals. More specifically, we were interested in finding out the perspectives of students about their learning processes and outcomes in the context of teaching the same discipline in the online medium, mediated by technology (in the 2020 cohort), and face-to-face *via* traditional classroom instruction (in the 2019 cohort). SWMG is a special compulsory discipline for undergraduate students in the second year of study, scheduled with 2 h of coursework and 2 h of workshops per week for 14 weeks. The learning objectives of SWMG for students were to acquire a set of knowledge, values, and skills to work with groups, to understand the main concepts and theoretical approaches regarding the use of groups in social work, to know and practise the main skills needed in working with groups, and to understand the particularities of working with different categories of beneficiaries of social work within groups. Developing social work practise competencies is complex and requires a combination of procedural competencies such as knowledge and skills, along with meta-competencies such as self-awareness and self-reflection (Kourgiantakis et al., 2020).

The theoretical and empirical frameworks on which we have based our research included: ways to define groups (Sampson and Marthas, 1981); concepts, values and skills in working with groups (Sampson and Marthas, 1981; Doel, 2006; Brandler and Roman, 2016); ecological influence on groups and group practise (Vinter and Galinsky, 1985; Tropman, 2004); types of groups by purpose: recreational groups, educational groups, support and self-help groups, socialisation groups, therapeutic groups and prevention groups (Kurtz, 2004; Nash and Snyder, 2004; Roffman, 2004; Zastrow, 2015; Toseland and Rivas, 2017); group dynamics (De Visscher and Neculau, 2001; Toseland et al., 2004); goals and stages of group development (Tuckman, 1963; Garland et al., 1976; De Visscher and Neculau, 2001; Shulman, 2016); tasks, roles, leadership and power within groups (Doel, 2006); online groups, virtual classroom and online support, online/technology-mediated teaching and learning (Gitterman and Salmon, 2009). The core of the *group work model* taught in the classroom was *the mutual aid model of social group work*. The main characteristics of this model have presented in the Schwartz (1961) definition of the social workgroup as: “an enterprise in mutual aid, an alliance of individuals who need each other, in varying degrees, to work on certain common problems. The important fact is that this is a helping system in which the clients need each other as well as the worker. This need to use each other to create not one but many helping relationships is a vital ingredient of the group process and constituted a common need over and above the specific task for which the group was formed” (p. 19). And more specifically the teaching and learning was focused on *group work approaches related to purpose*: support groups, educational groups, growth groups, therapy groups, socialisation groups, self-help groups, and prevention groups.

As a *pedagogical strategy* underlying the teaching model, we have used the service-learning/community-related service-learning framework. *Service-learning* is an innovative pedagogy that intertwines community service with the main objectives of a course from a reflective perspective, where the three stakeholders—students, faculty facilitators, and community members—engage in a mutual learning environment (Salam et al., 2017). In the triad mentioned above, community members represent the service recipient, faculty instructors perform as facilitators, organisers, and coordinators between the academic institution and the local communities, and students are engaged in service-learning experiences as service providers and learners (Salam et al., 2019, p. 585).

Higher education institutions can contribute to the development of local communities through collaboration with other organisations (Salam et al., 2019), including institutions and non-government organisations (NGOs) from the social work field. This organisational alliance “provides cognitive and psychological support” for different categories of vulnerable people and addresses different community problems: “Developing a program to help women re-enter the workforce; studying the social injustices in a community; investigating a social problem—ageism, sexual orientation, discrimination—and developing solutions” are few examples (Rutti et al., 2016, p. 429). Although community members need to devote their time and available resources for service-learning projects, their efforts are rewarding for the community as a whole. Service-learning projects can improve the overall well-being of the community (Salam et al., 2019).

From a theoretical perspective, a generalised framework for service-learning pedagogy has not been agreed upon yet among the scholars; however, most service-learning and community-related service-learning frameworks have roots in the experiential learning theory of Dewey (1938), further developed by Kolb (1984) in his experiential learning cycle, consisting of four phases: concrete experience; reflective observation, abstract conceptualisation, and active experimentation (apud Salam et al., 2019).

By getting involved as active participants in community learning projects, students acquire crucial and diverse skills, such as communication skills, problem-solving skills, analytical thinking, innovative solutions, and work in a collaborative environment (Salam et al., 2019). Community service-learning projects also challenge the values of students, increase their social awareness and responsibility, and strengthen their character and civic engagement (McLeod, 2013; Marshall et al., 2015).

Faculty members are at the centre of initiation through the design of service-learning courses and projects by ensuring smooth delivery of activities in the local communities and organisations (Salam et al., 2019). Along this process, professors play major roles as collaborators, facilitators, and supervisors who assist and monitor students in implementing their service or community learning projects (Voss et al., 2015).

*The purpose* of our research was *to find out the perceptions of students* about their *learning processes and outcomes* of SWMG, in the context of teaching the workshops *exclusively in the online medium*, due to the forced shift generated by the pandemic by comparison with the teaching of the same discipline in the *face-to-face environment via traditional classroom* instruction. The *main hypotheses* of the study are the following: (1) The teaching and learning mediums shape the type of skills achieved by students; and (2) General self-assessment of SWMG workshops in terms of their usefulness for field practise in social work is different according to the teaching and learning environment. For further study, our research also included several *questions*: (1) How important was the feedback provided by the coordinating teacher and by peer colleagues during SWMG workshops as a mode of learning? (2) What were the social problems, client populations, and types of groups chosen by the students to acquire the skills needed to lead a professional group in social work? (3) Which one of the social roles played by the students during the SWMG workshops brought them the greatest satisfaction and why? What social roles played by the students during SWMG workshops have they brought them the most discomfort, and why?

## Materials and Methods

### Study Design and Participants

This study has a mixed research design. It provides a perspective over 2 academic years, between 2018 and 2020. It attempts to investigate two different learning environments during the workshops of the compulsory discipline SWMG: the face-to-face environment vs. the online environment. The research focuses on the activities and deliverables of all the students that graduated the SWMG discipline in/ during the two academic years and that had the same coordinating teacher: 50 students in 2020 (cohort A) and 92 students in 2019 (cohort B). From the varied kinds of deliverables of students, like the final self-assessment sheet, the group plan, the laboratory protocols, lecture reading sheets, and other different homework. From all of these, the present study analyses the first two deliverables, using descriptive, inferential statistics and thematic content analysis.

### Ethical Issues

The data used in this study include the deliverables accomplished by the students during a semester for the workshops of the compulsory course SWMG. Since this study was designed after the evaluation of the students at this seminar, the researcher had to ask for the consent of the students to use the information from their deliverables after the data were gathered. The informed consent of the students was easily obtained because the researcher was also the coordinating teacher of the seminar. The students understood the usefulness of this study and showed their openness and confidence that the authors would respect the principles of confidentiality and anonymity regarding their data. The authors ensured the participants that if there were drawn out relevant excerpts from their final self-assessment sheets and the plans of the group, some photos from the group activities or some screen captures with certain online activities, their names or faces would be protected. The authors have provided information about the study at the end of a course where the two cohorts of students participated. A separate folder has been created on the platform Google Classroom, and the students uploaded the written and signed informed consent with their agreement to participate in the study. The study was approved by the Ethical Committee of the university affiliated with the authors.

### Procedure

In the second semester of the second year of study, students at the Social Work specialisation have to attend the compulsory discipline SWMG, summarising 14, 2-h long workshops. The main task of students at this workshop is to prepare and conduct a one-session group, working in pairs as co-leaders and involving their colleagues as group members with different assigned roles. The workshops led by students were initiated during the fourth week of the semester, after the coordinating teacher led three introductory workshops: an introductory seminar with sociometric games and icebreaking exercises, adequate for the initial stage of a group program; a second workshop that presented the focus groups as a research tool in to distinguish it from the intervention groups in social work; the third seminar providing a demonstrative group session conducted by the teacher on a theme related to the educational system in Romania, with diverse activities and techniques suitable for an educational type of group.

After these 3 introductory weeks, students began to implement their own group sessions. In 2019, when the learning medium was exclusively face-to-face, the two group co-leaders could arrange the classroom. They provided their colleagues the necessary class materials for their practical activities: white A4 papers, coloured pens, scissors, paper glue, post-it, and scrapbooking materials. In 2020, students had to adapt their practical group activities to the exclusively online workshops (see Annex 1 for some examples).

The students organised in pairs were given 45 min to lead a single-session group with their colleagues. In each workshop, two demonstrative group sessions were conducted. In searching the theme for their practical group session, students were encouraged to get the greatest possible advantage from their previous and current internship stages in the institutions and NGOs in the social work field from the communities of origin, mostly in the rural areas, or from Cluj-Napoca. In this municipality, the “Babeş-Bolyai” University is located. The field practise allowed the students to meet different vulnerable categories of beneficiaries and see how the professionals resolve their problems and fulfil their needs. Until the second academic year, students already learned theories and methods applied in the social work field with individuals and families. At the SWMG workshops, students were asked to conduct a one-session group, bringing a socio-psychological problem of a community in a practical scenario. Guided by this recommendation, students freely chose the social problem they were interested in, the client population, and the group type. The only request was to prepare a planning list and avoid the same combination of the three items. The rest of the students played each time the specific roles proposed by the co-leaders, according to the client population the group co-leaders proposed. After each group session, oral feedback with the co-leaders was given by their colleagues who participated as group members and by the teacher, who played the role of the passive observer during the group sessions. Before each group session, the two co-leaders had to design a plan for the forthcoming practical activity. The request was that the co-leaders should propose at least four activities and divide them equitably between themselves during their group session. Each group led by the students was audio or video recorded with the agreement of the audience, and the material was used only by the co-leaders to complete their group plan.

The task of planning and conducting a one-session group was designed from a service-learning perspective. During a semester, the students were allowed to use the knowledge from previous disciplines and the skills acquired in the internship stages to test a group approach to solve a community problem. They were also allowed to prepare themselves throughout a guided academic context to implement such group models in a real community environment during the third year of study when they chose their graduated thesis topic. The thesis of students in social work is done with the coordination of an academic adviser from the university and under the supervision of a professional from an institution or an NGO from the social work field. This pedagogical perspective corresponds to some frameworks described in the literature devoted to community-related service-learning programs (Ali et al., 2012; Salam et al., 2019). The implemented group session and the completed group plan counted two points from a total score of 4 points assigned to the evaluation criteria of the workshop.

During the academic year 2018–2019, the SWMG workshops unfolded in a classroom at the campus of the university, while in the second semester of the academic year 2019–2020, all the workshops, including the groups led by students, ran on the *Zoom* platform. In 2019, all the homework of students were delivered in print, as opposed to 2020, when all the deliverables that the students had to develop/ handle have been uploaded on the platform *Google Classroom* (see Annex 2 for an exemplification of the structure of deliverables students from cohort A had to follow). The teacher created a folder for each deliverable that the students had to upload, including the task requests, some explanation regarding the deliverable, and the deadline.

At the end of the semester, all students had to complete the final self-assessment sheet, an evaluation tool with reciprocal benefits. It was designed to help the students reflect on the overall experience and gains made by their involvement as leaders and group members with different social roles and provide the coordinating teacher valuable feedback about the perceptions of students of the efficiency of the practical activities and assignments. The self-assessment sheet had to be sent to the teacher during the week after the semester. It counted 0.5 points from a total of four points from the SWMG workshop. This self-assessment sheet served as an instrument for the instructor to refine the pedagogical strategy of the workshop to maximise the benefits of the professional acquisitions for students.

### Measurements

To respond to the objectives and questions of the study, we analysed two deliverables from the students: the single-session group plan and the final self-assessment sheet.

The group plan included the following compulsory sections: the psycho-social problem, the client population (role of group members), the type of group by purpose, the teaching techniques implemented during the workshop (the debate, the role-play, the exercise, the narration, etc.), the materials and technical means used (paper, pencils, whiteboard, post-it notes, a projector, a computer, short videos from the internet, presentation of the information in PowerPoint), and a short description of each activity proposed by the co-leaders with the contribution and feedback of the group members and of the evaluation of the teacher. The co-leaders were given 1 week to finish this assignment by incorporating some relevant contributions of the activities of the colleagues of a group and a qualitative evaluation for each of the four group activities and then to provide the teacher the completed plan of the group session.

The self-assessment sheet included qualitative and quantitative measures related to the diversified activities of students accomplished during the SWMG workshops. Although not conceived as a standardised tool, the self-assessment sheet had more sections dedicated to: the protocols containing a reflective work on two self-selected workshops; the roles played in the group sessions as leaders and as different group members; a reflection on the feedback received and given from the perspectives of leaders and group members; gains from the overall participation at the workshops; the relevance of the literature they read; the evaluation of the general usefulness of the workshops; qualitative feedback regarding the usefulness of the final self-assessment sheet and the SWMG workshops as a whole.

From the structure of the self-assessment sheet, we decided to analyse four items from different sections. First, we were interested in the types and number of skills students declared they acquired during the SWMG laboratory. These indicators were captured through the following question: “In which areas do you think you benefited the most from participating in SWMG workshops?” In the sheet, the following examples were given from which students could choose one or more: self-awareness; communication; networking; reflection/ conceptualisation of experiences; self-control; improvement of other professional skills. For this study, we grouped these skills into three categories: (a) training of cognitive or conceptual skills and self-awareness; (b) training of interpersonal skills; and (c) improvement in other professional skills. Students could choose one skill, more skills, or no skills; and if the last category was selected, students were invited to elaborate.

At the end of the self-assessment sheet, students were asked to evaluate the overall usefulness of the workshop. A Likert scale was used for this item, with 6 points from 1, meaning totally useless, to 6, meaning very useful.

From the four items dedicated to the feedback, we selected the last one for the analysis, which was formulated as follows: “As a way of learning, I consider that feedbacks are: as important as the roles played; less important; not at all important; I do not know.”

We gave special attention to evaluating the roles played by students as members of the group sessions led by their colleagues. In particular, an item with two questions was taken into account for this study. Students were asked to reflect and to write about the following personal experiences: “Choose 3 roles that brought you the greatest satisfaction and explain the source of satisfaction (I managed to get into the role, the role made me more sensitive, the role raised my awareness, through the role I learned new things)?”; and “Choose 2–3 roles that made you uncomfortable and explain the source of dissatisfaction (I did not manage to get in the role, I was asked too much, I did not know what to do in the role, I was not interested, or other reasons)?”

### Data Analysis

To fulfil the purpose of the study, to answer the research questions, and to test the formulated hypothesis, we applied the techniques of descriptive and inferential analysis.

The answers collected from two deliverables of the students (group session plans and final self-assessment sheets) were included in two correspondent SPSS databases (one for each deliverable). Frequency tables and frequency percentage distributions were generated in the descriptive analysis. Significance tests specific to inferential statistics, namely Independent-Samples *T*-Test and association, were used for testing the hypotheses after the variables' classes and values were recoded to meet the criteria of each test applied.

## Results

In this section, we present the results of our analysis from a comparative perspective by the learning medium of the two investigated cohorts: the online learning medium for the 2020 cohort (cohort A) and the traditional face-to-face classroom learning medium for the 2019 cohort (cohort B).

Based on the analysis of data from the self-assessment sheet, the answers of the students at the item related to the achieved skills generated a sub-set of classes for each category, taking into account that the students also indicated a combination of the acquired skills as shown in Table 1.

**Table 1:** Descriptive statistics on the skills students declared they acquired through the participation at the social work methods with groups workshops by cohort/learning environment of students.

**Category of skills**	**Students' cohort/learning environment**			
	**2020 – online**		**2019 - face-to-face**	
	***N***	**%**	***N***	**%**
**Training of cognitive/conceptual skills and self-awareness**				
None	7	14.0	26	28.3
Reflection/conceptualisation of experiences	10	20.0	12	13.0
Self-awareness	19	38.0	34	37.0
Both reflection of experiences and self-awareness	14	28.0	20	21.7
*Total*	50	100	92	100
**Training of interpersonal skills**				
None	11	22.0	11	12.0
Communication	13	26.0	20	21.7
Networking	6	12.0	11	12.0
Communication and networking	15	30.0	36	39.1
Communication and networking and self-control	5	10.0	14	15.2
*Total*	50	100	92	100
**Improving of other professional skills**				
None	27	54	38	41.3
Improvement of other professional skills	23	46	54	58.7
*Total*	50	100	92	100

According to Table 1, participation at the SWMG workshops in the online medium favoured the *training of cognitive/conceptual skills and self-awareness* to a greater extent than in the face-to-face learning environment. The opposite is true when the *improvement of other professional skills* is concerned. The distribution of interpersonal skills training presents variations across the cohorts: communication skills prevail in cohort A, while the combination of interpersonal skills is more frequently reported in cohort B. The high percentage of students who perceived no learning of interpersonal skills during the online group sessions is a striking result. Networking recorded the same relative weight in the list of interpersonal skills in both cohorts.

We summed up the skills mentioned by the students by category to test the first hypothesis, assuming that the environment of learning shapes the type of skills acquired by the students. The first category of skills acquisition had three possible values: 0 for none; (1) for either reflection/conceptualisation of experiences or self-awareness; (2) for both reflections of experiences and self-awareness, respectively. The second category of skills acquisition had four possible values: 0 for none; (1) for either communication or networking; (2) for both communication and networking; (3) for the three skills mentioned together (communication and networking and self-control). The third category had: 0 for none and 1 for mentioning one of the other professional skills (e.g., empathy, active listening, and assertiveness). We used the Independent-Samples *T*-Test successively. We had an independent grouping dichotomous variable, represented by the two cohorts of students, and a quantitative dependent variable, measuring the number of acquired skills for each of the three investigated categories.

As Table 2 shows, there is a significant difference between the two cohorts of students regarding interpersonal skills learning. The results of the Independent-Samples *T*-Test illustrated in Table 2 indicate that in cohort B, students reported that they learned the interpersonal skills to a significantly greater extent than in cohort A (*t* = 2.372, *p* < 0.05). There is no significant difference in learning cognitive/conceptual skills and self-awareness and improving other professional skills between the two cohorts of students.

**Table 2:** The *t*-test results reflecting the differences between the number of skills reported by students according to the year/learning environment and the category of skills.

**Dependent variable**	**Cohort**	**Descriptives**		***T*** **-test result**
Training of cognitive/conceptual skills and self-awareness	2020	*N* Mean St. deviation	50 1.16 0.65	*t* = 1.862 df = 140 *p* = 0.065
	2019	*N*	92	
		Mean	0.93	
		St. deviation	0.71	
Training of interpersonal skills	2020	*N* Mean St. deviation	50 1.30 0.93	*t* = 2.372df = 140*p* = 0.019
	2019	*N*	92	
		Mean	1.67	
		St. deviation	0.88	
Improving of other professional skills	2020	*N* Mean St. deviation	50 0.50 0.58	*t* = 1.034df = 140*p* = 0.303
	2019	*N*	92	
		Mean	0.60	
		St. deviation	0.52	

The second hypothesis assumes that the general self-assessment of SWMG workshops regarding their usefulness for professional/field practise in social work is different according to the learning environment.

The result of the association, illustrated in Table 3, reflects a significant difference in terms of the general perception of the usefulness of SWMG workshops between the two cohorts of students (χ^2^ = 8,591, *p* < 0.05). The distribution of frequencies indicates that cohort A reported to a greater extent that the SWMG workshops were very useful compared to cohort B (78 vs. 53.3%).

**Table 3:** The general self-assessment of social work methods with groups workshops in terms of their usefulness for field practise by cohort/learning environment.

**Degree of SWMG workshops' usefulness**	**2020**		**2019**		**Result of association**
	***N***	**%**	***N***	**%**	
Very useful	39	78.0	49	53.3	χ^2^ = 8,591
Useful	6	12.0	27	29.3	df = 2
Some useful, some useless	5	10.0	16	17.4	*p* = 0.014
Total	50	100	92	100	

We looked at the importance that the students attached to the feedback they received from their colleagues and their coordinating teacher as a way of learning for the co-leading role and the other roles related to different client populations they played during the semester.

As shown in Table 4, almost 90% of all students appreciated that the feedback they received from the coordinating teacher and their colleagues during the SWMG workshops was as important as their roles in the groups they attended.

**Table 4:** The importance of feedback as a way of learning.

**Cohort**	**Importance of feedback students received from the coordinating teacher and from their colleagues**					
	**As important as the roles they have played in the groups that they have attended**		**Less important than the roles they have played in the groups that they have attended**		**Total**	
	***N***	**%**	***N***	**%**	***N***	**%**
2020	45	91.8	4	8.2	49	100
2019	76	88.4	10	11.6	86	100
Total	121	89.6	14	10.4	135	100

Analysis of the group plans developed for the SWMG workshops conducted by students from both cohorts included three dimensions/items: social problem or issue; client population; and type of group by purpose.

Table 5 presents the social/psycho-social problem or issue selected by students that conducted SWMG workshops in the online learning environment in 2020 and presents comparisons with students that conducted them in the face-to-face environment in 2019. Summarising the preferences of students, we can notice that social problems prevail in both cohorts, with a higher rate among students from cohort B than among those from cohort A (68 vs. 56%). Students who led their group work in the online environment preferred issues from the psychological spectrum compared with those who conducted session groups face-to-face (44 vs. 32%). A look at the sub-categories of this type for the 2020 cohort reflects the preoccupation of students with topics related to social isolation in time of the pandemic, affecting self-esteem, psycho-emotional balance, or the capacity to cope with certain social statuses.

**Table 5:** The psycho-social problem/issue elected by students who conducted Social Work Methods with Groups workshops in the online learning environment−2020 and 2019 cohorts.

**The category of psycho-social problem/issue**	**Sub-categories (*N*, 2019–2020)**	***N*** **/% by sub-category**	
		**2019**	**2020**
Social	discrimination—of Roma people (2-0), against women in accessing the labour market and at the workplace (1-0), at the workplace (1-0), of children with disabilities (0-1), racism (1-0), in general (1-0); addiction or excessive consumption—of alcohol (2-0), drugs (3-2), gambling (1-0), work (1-0); bullying—cyberbullying, labelling among pupils (4-0); violence—in school (1-1), domestic (2-2); marginalisation of refugees (1-0); poverty and social inequality (1-0); child abuse (1-0); effects of institutionalisation on children (1-0); volunteering—its importance for youth, training of volunteers to integrate in an NGO team and for intervention in a Roma community (3-0); the problems of Romanian medical system (1-0); loneliness—and social isolation, in time of pandemic (2-3); difficulties of coping with new social status—divorced, single motherhood (0-2); risk of relapse (0-2); sexual education in schools (1-0); abortion (1-0); school dropout (0-1); bonus and malus of new epidemics Covid-19 (0-1)	32/68	15/56
Psychological/psycho-social	low self-esteem and lack of self-confidence (1-6); low self-awareness (0-2); memory disorders in old age (1-1); psycho-emotional imbalance (0-3); low level of assertiveness (1-0), importance of—mutual knowledge in a new group of pupils (2-0), a positive self-image and of awareness of self-worth (1-0), of feedback from peer group (1-0), harmonious social relations (1-0), friends in youth's life (2-0), childhood in adulthood (1-0), recreational games for children (1-0); role of parents in children's education (1-0); communication problems caused by disability (1-0); well-being (1-0); socio-emotional needs of people with visual impairments (1-0)	15/32	12/44
Total (N/%)		47/100	27/100

*In the second column, in the round brackets, you can see the number of group sessions by specific topics conducted in each year: the first number refers to the number of topics chosen by students from 2019 cohort, and the second reflects those topics elected by students from 2020 cohort. The last line of this table indicates the total number of group sessions conducted in 2019, respectively in 2020*.

In analysing the client population the co-leaders addressed in their SWMG group session, we organised the data according to these main life stages: childhood, adolescence, youth, adulthood, and old age. Tables 6A,B address the situation in cohorts A and B, respectively.

**Table 6A:** The client population the co-leaders addressed in their social work methods with groups (SWMG) workshops in 2020 cohort A.

**Client population/group members' roles in the SWMG workshops**		***N***	
**Category**	**Sub-category**	**By** **sub-category**	**By** **category**
Children	Who witnessed domestic violence	1	2
	Who failed at school	1	
Adolescents	In general (aged 14–18 or 16–18)	3	3
Young people	Students (in general or before the exam session)	4	14
	Isolated at home	3	
	Single mothers	3	
	Living in a residential centre from the child protection system	2	
	Former consumers of ethnobotanicals	1	
	Refugees	1	
Adults	With low self-esteem	1	6
	With psycho-emotional imbalance	1	
	Recently separated/divorced	1	
	Women victims of domestic violence	1	
	Parents of children with disabilities	1	
	Persons who have committed an infraction while driving in the context of alcohol consumption, under supervision in the probation service	1	
Old people	Over 65 years, retired	1	2
	Lonely old people	1	
Total of group plans			27

**Table 6B:** The client population the co-leaders addressed in their social work methods with groups (SWMG) workshops in 2019—cohort B.

**Client population/group members' roles in the SWMG workshops**		***N***	
**Category**	**Sub-category**	****By **sub-category**	****By **category**
Children	Primary school students	4	7
	5th grade students	1	
	With special needs	1	
	In a day centre who excessively use the mobile	1	
Adolescents	High school students (9th to 12th grade)	14	17
	12th grade from a community where a group of refugee is about to be integrated	1	
	Single mothers under 18 years	1	
	In general (aged 17–19)	1	
Young people	Students themselves	10	18
	Students in the last year of study	2	
	Volunteer students	3	
	Students in the 1st year studying pedagogy	1	
	Whose parents migrated abroad for work	1	
	In the role of future parents	1	
Adults	Alcohol dependent	1	1
Old people	In general	1	2
	Over 70 years	1	
Mixed	Former patients and medical staff from Romanian hospitals	1	2
	Parent-child dyad	1	
Total of group plans			47

Regarding the client population chosen by students in cohort A for their group sessions, 10 from 27 groups (33.33%) addressed the category of young people: students themselves (four groups), isolated at home (three groups), living in a residential centre from the child protection system (two groups) and former consumers of ethnobotanicals (one group).

In cohort B, students opted for themselves (10 groups) and volunteer students (three groups), meaning 13 groups, respectively, 27.66% from a total of 47 group sessions.

Consequently, there is a slight difference between the two cohorts (of 5.67%), indicating that students were more prone to play their own role in the group sessions during the pandemic. Looking at the adult category, a remarkable observation is that the students from cohort A preferred a typology of people with psycho-emotional problems (five groups).

In both cohorts, there was a propensity for students to choose age categories similar to their life stage: youth and adolescence. Interestingly, students directed their attention to children and the elderly to the lowest extent.

The analysis of group types by purpose is illustrated in Table 7 for both cohorts of students. The relative distribution reflects different preferences among the students by cohort. In cohort B, the students mostly preferred the educational (46.8%), prevention (21.3%), and socialisation (14.9%) groups, and they had the least preference for therapy and support groups. In cohort A, the students tended to choose group types that were associated with psychological outcomes. Thus, support (44.4%), growth (22.2%), and therapy (11.1%) groups were their most preferred groups.

**Table 7:** Type of group by purpose−2019 and 2020 cohorts.

**Type of group**	**2019**–**Cohort B**		**2020**–**Cohort A**	
	***N***	**%**	***N***	**%**
Support group	2	4.3	12	44.4
Educational group	22	46.8	4	14.8
Growth group	4	8.5	6	22.2
Therapy group	1	2.1	3	11.1
Socialisation group	7	14.9	0	0
Self-help group	1	2.1	0	0
Prevention group	10	21.3	2	7.4
Total	47	100	27	~100

**Figure 1:**
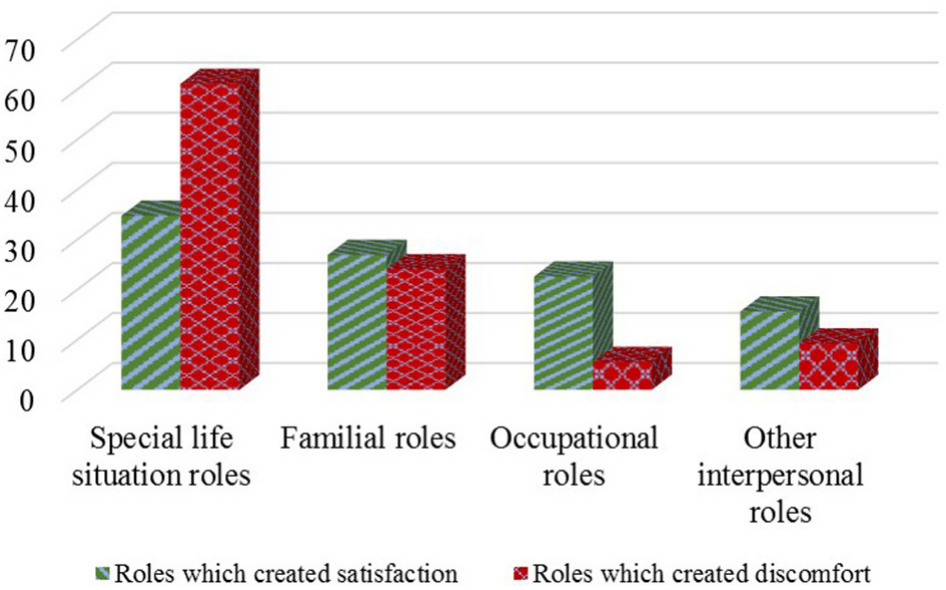
The distribution of roles played by students during the Social Work Methods with Groups workshops in the online learning environment.

The last analysis relates to the perception of the roles played by the students as co-leader and as group members that produced satisfaction or discomfort. In the final self-assessment sheet, the students were asked to enumerate and briefly describe three roles that satisfied them and two to three roles which created personal discomfort. The results are presented in Figure 1.

According to Figure 1, the level of satisfaction was higher in the case of occupational and other interpersonal roles, and discomfort was more prevalent when special life situation roles were interpreted. Familial roles produced quite similar levels of satisfaction and discomfort.

## Discussion and Conclusions

The results of our study showed some relevant distinctions between the perceptions and experiences of the two analysed cohorts of students, which were shaped by different learning environments: entirely face-to-face in 2019 (cohort B) and exclusively online in 2020 (cohort A). Students who delivered their group sessions during the pandemic in 2020 perceived that they had trained *especially the cognitive/conceptual skills and self-awareness*, while students who conducted their group sessions in a traditional face-to-face classroom reported they exercised, in particular, *the interpersonal skills* (to aid in mutual understanding and affective skills) and *other professional skills, like contractual and problem-solving skills*.

According to the teaching/learning medium/environment, there is a significant difference in the general perceptions of the students from the two cohorts that were investigated regarding *the usefulness of SWMG workshops for professional practise* in the social work field. About three-quarters of students from the online learning environment reported that the SWMG workshops were useful or useful compared to slightly over half of students from the face-to-face learning environment. The fact that students perceived greater usefulness of the SWMG workshops in the online environment could be explained by at least two additional ingredients of the online workshops: *the therapeutic effect of the communication in the group setting* as an alternative to the isolation and the use of technology as a tool of learning, not as an amusing device that diverts the attention from teaching, as was the case in the face-to-face learning environment. Almost 90% of all students appreciated that the feedback they received from the coordinating teacher and their classmates during the SWMG workshops was a valuable way of learning, and this was as important as the roles they played in the groups they had attended. *The online instruction slows down the learning process*, as the students must limit their questions to essentials/short descriptions and then give the teacher and the colleagues time to respond.

The results related to the choices of students for the psycho-social problem and the more specific themes for their group sessions reflect the diversity of institutions and NGOs from the social work field where the students completed their academic internships on the one side. The concerns of the students for a great variety of socio-psychological issues manifested in the Romanian society, among which domestic violence and violence against women, issues related to risky behaviours in young people (Faludi and Rada, 2019), and different problems of social functioning of diverse, vulnerable groups in interaction with their living environment (Neamţu, 2001; Gunzl and Neamţu, 2012). A greater appetite for these types of problems characterised the 2019 cohort when students were able to do internships in a variety of institutions and NGOs, while in 2020, students who were forced to stay at home preferred to focus on therapeutic and support groups, as a response to the constraints imposed by restrictions and affected by lack of academic contacts and geographical isolation. The strategy used in the planning and implementation of the groups used in the pedagogical strategy of the SWMG workshop in the two consecutive academic years could serve as a model for other schools of social work who want to develop the same social work skills described in this study.

Remote learning is not the most desirable educational option for a variety of reasons. If technical problems occur, equipment failures can interfere with virtual group participation, and online students may not communicate, submit their assignments, or access the study materials. Ideally, the social work field students gain the most from the hybrid teaching-learning vehicles: face-to-face *via* traditional classroom and online media, which can be combined in many creative ways. Both ways proved to be relatively effective. The next question is if one way is truly better than the other and from which perspective? Blended learning approaches show promising outcomes for teaching social work methods as the students can learn about cognitive/conceptual skills online and apply this knowledge in the classroom.

A blended or hybrid social work lab can take full advantage of the benefits of each platform—online and face-to-face –to promote student learning better than either platform alone (Jackson et al., 2020). One of the most important advantages of blended learning for students is to acquire a multitude of real-world skills that directly translate into life skills, preparing the students for their future by using social group work (Golea and Neamţu, 2012). “All students no matter their age learn differently and teaching methods should reflect this, by designing teaching programs in a way that reaches visual, auditory, and kinetic learners alike. With the heavy integration of technologies, we'll be able to improve teaching, information retention, engagement, responsibility, and enjoyment. Students never outgrow their learning styles, meaning blended learning is more important than ever, no matter what the industry is, from schools to corporations, from all walks of life” (TeachTaught., 2021). The research of Garrison and Kanuka (2004) concludes that blended learning has the proven potential to enhance the effectiveness and efficiency of learning experiences.

The significance of our study is mainly in the area of comparative assessment of learning effectiveness from the perception and perspective of students, when studying the same discipline in the social work field, using the approach of service-learning as a variety of community-based learning in the context of two different teaching and learning environments and media: online and face-to-face instruction. Depending on what knowledge, skills, and competencies we wish to be acquired as outcomes of the social group work learning, we can choose a strategic combination of components focusing on modalities that students perceive as helpful and avoiding modalities that students perceive as not useful. Teaching SWMG role-playing can provide opportunities to understand various sides of a social problem and understand other viewpoints, and it can be effectively used in-person and virtual classes. It is important to provide guided self-reflection and supervision in a trusting learning environment for the effectiveness of the learning of students. Online relationship building and teaching the use of self in the virtual space can create major challenges regarding the effectiveness of social work education. To face the reality of uncertain times in an adaptative manner, the teaching-learning process in social work education needs innovation for the development of skills and competencies relevant for the society of the future. Our work opens new horizons for higher education delivery in applied social and behavioural sciences and reflections on future relations between teaching, research, and social service learning.

## Data Availability Statement

The raw data supporting the conclusions of this article will be made available by the authors, without undue reservation.

## Ethics Statement

The studies involving human participants were reviewed and approved by Ethics Committee of Babeş-Bolyai University. The patients/participants provided their written informed consent to participate in this study.

## Author Contributions

All authors listed have made a substantial, direct and intellectual contribution to the work, and approved it for publication.

## Conflict of Interest

The authors declare that the research was conducted in the absence of any commercial or financial relationships that could be construed as a potential conflict of interest.

## Publisher's Note

All claims expressed in this article are solely those of the authors and do not necessarily represent those of their affiliated organizations, or those of the publisher, the editors and the reviewers. Any product that may be evaluated in this article, or claim that may be made by its manufacturer, is not guaranteed or endorsed by the publisher.
