# Supplementary material for: Learning Effectiveness of Social Work Methods With Groups, in Online and Face-to-Face Contexts
Source: Front Psychol. 2021 Aug 17;12:649691. doi: 10.3389/fpsyg.2021.649691 (PMC8418085; doi:10.3389/fpsyg.2021.649691)

ANNEX 1. Some photos reflecting the group sessions of students from cohort B and A

*A face-to-face group session for people with visual impairments in 2019 (cohort B)*

*
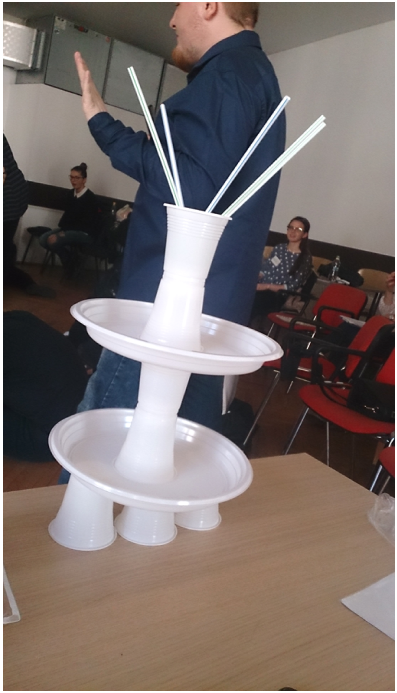
*


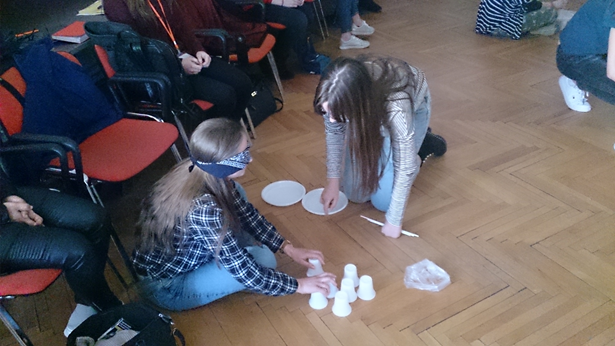


*A face-to-face group of reminiscence for elderly in 2019 (cohort B)*


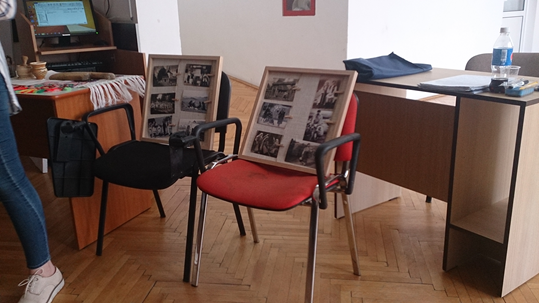


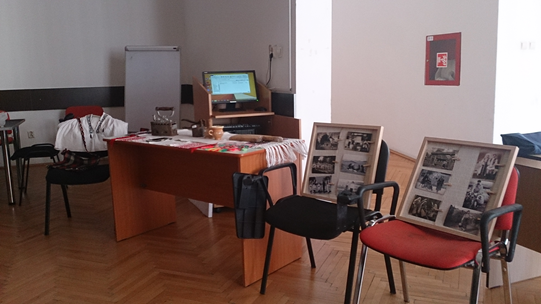


*Selection of slides from a Power Point presentation from an online one-session groups – social problem (domestic violence), client population or group members’ role (children who witness domestic violence), type of group (therapy group) in 2020 – cohort A*


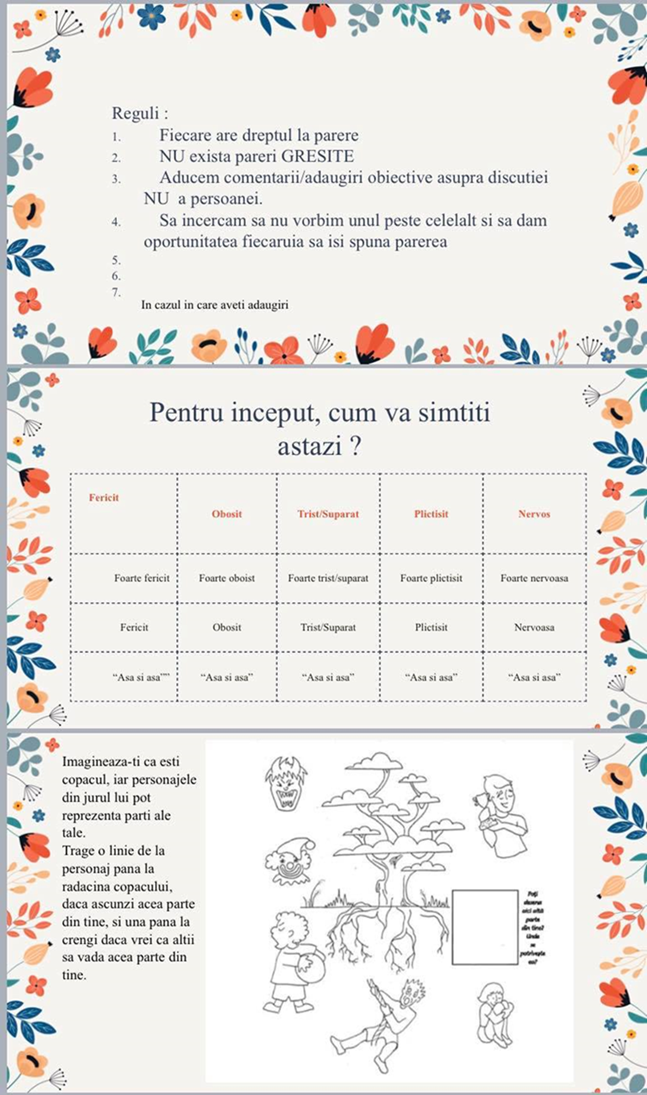


*The whiteboard where the group members expressed their ideas in the context of the Game of lines, from an online one-session groups – social problem (the psycho-social imbalance before the exam session), client population or group members’ role (students themselves), type of group (therapy group) in 2020 – cohort A*


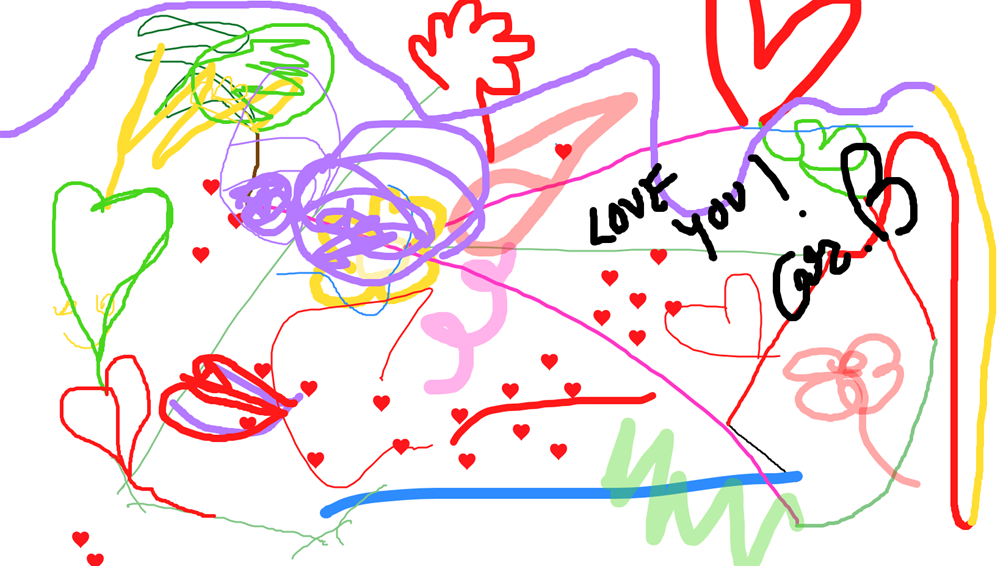


The game requirements:

On the whiteboard you have at your disposal, start to draw a line; each of you should continue in the way you want the line started by the other ones. Use different colors. The last participant ends the game, namely the line. Then name the image that resulted from everyone's cooperation. Motivate the chosen title.

Some students’ interpretation:

Examples: "I think it shows how different we are", "an amalgam of thoughts and feelings", "we can see that many of us made hearts, popcorn, romantic things", "and used warm colors", "it might be a diary of our current state"," I would say it shows a close connection between us".

**ANNEX 2. The structure of the Google Classroom for the discipline Social Work Methods with Groups – the structure of the section dedicated to the students’ deliverables in the time of pandemia in 2020 – cohort A**


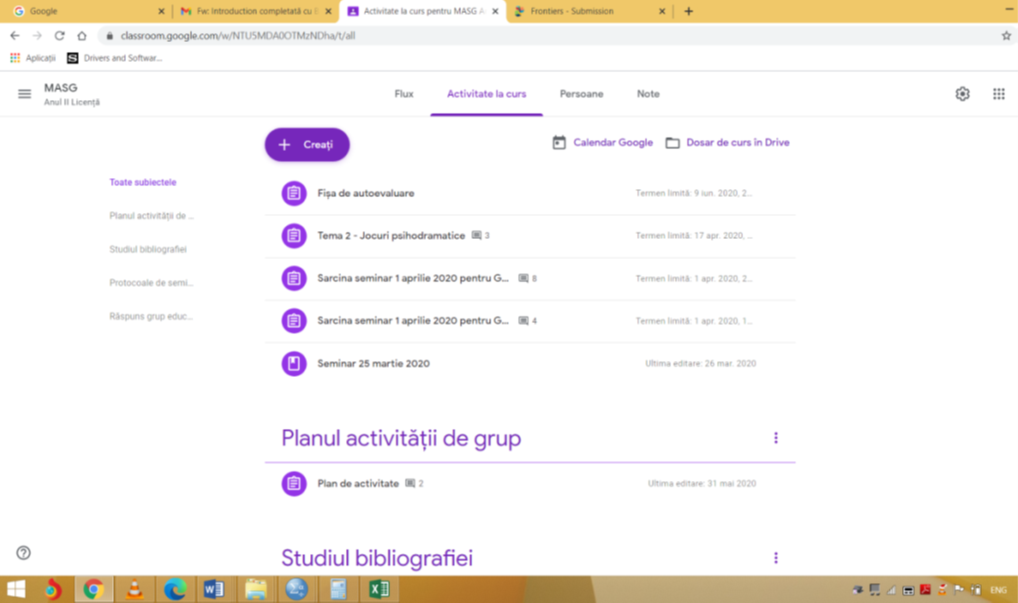

Supplement: Supplementary file 1 [file Data_Sheet_1.docx]
